# Association of Age-Related Macular Degeneration With Risk of All-Cause and Specific-Cause Mortality in the National Health and Nutrition Examination Survey, 2005 to 2008

**DOI:** 10.1001/jamaophthalmol.2018.6150

**Published:** 2018-12-20

**Authors:** Zhuoting Zhu, Wei Wang, Stuart Keel, Jian Zhang, Mingguang He

**Affiliations:** 1 State Key Laboratory of Ophthalmology, Zhongshan Ophthalmic Center, Sun Yat-sen University, Guangzhou, China; 2 Centre for Eye Research Australia, University of Melbourne, Royal Victorian Eye and Ear Hospital, East Melbourne, Australia

## Abstract

**Importance:**

Age-related macular degeneration (AMD) is the leading cause of irreversible visual impairment and blindness in the United States if subretinal neovascularization is left untreated. Knowledge of the association between AMD and survival is informative for underlying mechanisms of AMD.

**Objective:**

To examine the association between AMD and risk of all-cause and specific-cause mortality in a representative US sample.

**Design, Setting, and Participants:**

This population-based prospective cohort study included 5603 participants 40 years or older who responded to the National Health and Nutrition Examination Survey (NHANES) in the 2005-2008 phase. Retinal photographs were graded as early, late, or no AMD. All analyses accounted for the complex and stratified design of NHANES with weighted data. Risks of all-cause mortality were assessed with Cox proportional hazards regression models; risks of specific-cause mortality, with Fine and Gray competing risks regression models. Time to death was counted from baseline to date of death or December 31, 2011, whichever came first. Data analysis was conducted from April 1 through 30, 2018.

**Exposures:**

Age-related macular degeneration status as determined by digital fundus images.

**Main Outcomes and Measures:**

Mortality resulting from all causes and specific causes until December 31, 2011.

**Results:**

Among the 5603 participants (52.6% female [n = 2793] and 77.1% white [n = 3017]; mean [SE] age, 56.4 [0.4] years), weighted prevalence of any AMD was 6.6%, with 386 (5.8%) having early AMD and 55 (0.8%) having late AMD. After a median follow-up of 4.5 years (interquartile range, 3.6-5.6 years), 433 (5.3%) died of all causes, of whom 361 (83.1%) had no AMD, 54 (11.5%) had signs of early AMD, 18 (5.4%) had signs of late AMD, and 72 (16.9%) had any AMD at baseline. Overall, unadjusted all-cause and specific-cause mortality rates were higher for those participants who had early, late, or any AMD compared with no AMD. However, after adjusting confounding factors, only late AMD was associated with more than a doubling of all-cause mortality (hazard ratio [HR], 2.01; 95% CI, 1.00-4.03) and more than a 3-fold higher risk of mortality due to causes other than cardiovascular disease and cancer (HR, 3.42; 95% CI, 1.38-8.49). No association was identified between AMD presence or early AMD and all-cause or specific-cause mortality.

**Conclusions and Relevance:**

In this study’s findings, only late AMD was independently associated with all-cause mortality and mortality due to causes other than cardiovascular disease and cancer, indicating that late AMD may be a marker of biological aging. Alternatively, this association may be due to unmeasured or inadequately assessed confounding factors for late AMD.

## Key Points

**Question:**

What is the association of age-related macular degeneration with mortality outcomes among older adults?

**Findings:**

In this cohort study of 5603 participants 40 years or older, only late age-related macular degeneration was associated with more than a doubling of all-cause mortality and more than 3-fold higher risk of mortality not due to cardiovascular disease and cancer.

**Meaning:**

The association of late age-related macular degeneration and poor survival may shed light on mechanisms underlying the disease, indicating that late age-related macular degeneration may be a marker of frailty and aging or may be due to residual confounding factors indicative of aging.

## Teaser

This population-based cohort study examines the association between age-related macular degeneration and the risk of all-cause and specific-cause mortality in a representative US sample of older adults from the 2005-2008 National Health and Nutrition Examination Survey.

## Introduction

Age-related macular degeneration (AMD) is the leading cause of irreversible visual impairment and blindness in the United States if subretinal neovascularization is left untreated.^1^ The estimated annual direct health care expenditure due to AMD in the United States is more than $4.6 billion.^2^ The global incidence of AMD is projected to increase, with an estimated 196 million patients with AMD by 2020.^3^

Despite the heavy burden of AMD, mechanisms underlying AMD remain poorly understood. Previous epidemiologic studies have identified some consistent risk factors (smoking) and systemic comorbidities, including cardiovascular disease (CVD)^4,5,6,7,8,9,10,11^; however, results relating to the association between AMD and survival are conflicting.^12,13,14,15,16,17,18,19,20,21,22,23,24,25^ Speculation has suggested that these inconsistent results arise from inadequate adjustment of important confounding factors, for example AMD-associated systemic comorbidities (CVD) that may lead to poorer survival.^26^ Moreover, most previous studies^27^ might overestimate the absolute risk of CVD mortality by failing to account for a competing risk of death. Given the current concern that injection of anti–vascular endothelial growth factor (anti-VEGF) may lead to increased risks of thromboembolic events,^28,29^ knowledge of the accurate influence of AMD on mortality risk, especially CVD mortality, is warranted.

The National Health and Nutrition Examination Survey (NHANES) is an ongoing population-based study. This nationally representative sample of the noninstitutionalized US population provides an opportunity to investigate the association between AMD and all-cause and specific-cause mortality in the context of comprehensive demographic, health-related behaviors and comorbidities.

## Methods

### Sample and Population

Led by the National Center for Health Statistics of the Centers for Disease Control and Prevention, Hyattsville, Maryland, the NHANES adopts stratified multistage sampling methods. Details of the sampling and testing methods have been described in detail elsewhere.^30^ Briefly, participants undergo comprehensive health-related interviews and examinations every 2 years. The NHANES purposely oversamples participants older than 60 years and Hispanic and African American individuals. In adherence to the tenets of the Declaration of Helsinki, NHANES protocols were approved by the National Center for Health Statistics research ethics review board, and participants provided written informed consent.

### Retinal Photography and AMD Grading

During the 2005-2008 phase of NHANES, retinal images were collected among participants 40 years or older. An ophthalmic digital imaging system (CR6-45NM; Canon USA, Inc) and digital camera (EOS 10D; Canon USA, Inc) were used to capture retinal images. All fundus images were graded at the University of Wisconsin, Madison, according to the modified Wisconsin Age-Related Maculopathy Grading Classification Scheme.^31,32^ All images were graded by at least 2 experienced graders, with any disagreements adjudicated by a third senior grader. Early AMD was defined as signs of drusen with a grid area greater than a 500-μm circle and/or pigmentary abnormalities, whereas the presence of exudative or geographic atrophy signs was defined as late AMD. If retinal images were available for both eyes, the eye with the more severe status was used in the analysis.

### Mortality Data

Mortality data were derived from the 2015 public-access linked mortality archives. Mortality data were matched with files from the National Death Index via a probabilistic matching algorithm.^33^ A unique study identifier of the NHANES was used to link to the mortality data. All NHANES participants 18 years or older were available for mortality follow-up. Specific cause of death attributable to CVD was determined by codes I00 to I09, I11, I13, and I20 to I51 (diseases of heart) and I60 to I69 (cerebrovascular diseases) from the *International Statistical Classification of Diseases and Related Health Problems, Tenth Revision* (*ICD-10*). Cancer mortality was defined by deaths based on *ICD-10* codes C00 to C97. Those deaths not classified as CVD or cancer related were considered deaths due to other causes. Participants not matched with death certificates were considered alive. Time to death was counted from baseline to date of death or December 31, 2011, whichever came first.

### Covariates

A broad array of information regarding demographic factors and health-related behaviors and characteristics was assessed through in-person interviews and examinations. Specifically, age was categorized by 10-year age groups as 40 to 49, 50 to 59, 60 to 69, 70 to 79, and 80 years or older. Race/ethnicity was categorized as non-Hispanic white, non-Hispanic black, Mexican American, or other. Educational attainment was dichotomized as less than a high school diploma and a high school diploma or more. Marital status (unmarried or other vs married or living with a partner) was analyzed as a 2-level categorical variable. The indicator for family income (poverty income ratio) was classified as below poverty line (<1.00) or at or above poverty line (≥1.00). Smoking status was categorized as never, former, or current. Alcohol consumption was determined from participant interviews and divided as lifetime abstainer or former drinker, current drinker consuming no more than 3 drinks per week, and current drinker consuming more than 3 drinks per week.

Body mass index was calculated as weight in kilograms divided by height in meters squared and categorized as underweight (<18.5), normal to overweight (18.5-30.0), or obese (≥30.0). Diabetes was defined as self-reported physician diagnosis, use of diabetic tablets or insulin, or a glycosylated hemoglobin level of 6.5% or greater (to convert to a fraction of the total, multiply by 0.01). The presence of hypertension was characterized by self-reported history of hypertension, use of antihypertensive agents, or a systolic blood pressure of 140 mm Hg or higher and/or a diastolic blood pressure of 90 mm Hg or higher based on the mean value of 3 measurements. The presence of dyslipidemia was defined as a total cholesterol level of at least 240 mg/dL (to convert to millimoles per liter, multiply by 0.0259) or the use of a prescribed agent to lower cholesterol levels. As an indicator of vascular risk, the ratio of low-density to high-density lipoprotein cholesterol levels was calculated. A high level of C-reactive protein was defined as at least 1 mg/dL (to convert to nanomoles per liter, multiply by 9.524). A score of 10 or greater on the 9-item Patient Health Questionnaire (range, 0-27) was characterized as having depressive symptoms.^34^ The history of comorbid age-related ocular diseases, including cataract, glaucoma, and retinopathy, was based on the questionnaire and/or retinal images in accordance with previous studies.^35,36,37^ Walking disability was defined by self-report of difficult walking or need of special equipment for walking. Self-rated health status was dichotomized as poor to fair or good to excellent. Comorbid medical conditions included self-reported physician diagnosis of congestive heart failure, coronary heart disease, angina, heart attack, stroke, and cancer.

### Statistical Analysis

Data were analyzed from April 1 through 30, 2018. We combined the 2005-2006 and 2007-2008 phases of NHANES. All analysis accounted for the complex and stratified design based on NHANES analytic and reporting guidelines. Baseline characteristics of study participants, including age, sex, race/ethnicity, educational attainment, marital status, family income, smoking status, alcohol consumption, diabetes, hypertension, high cholesterol level, ratio of low-density to high-density lipoprotein cholesterol levels, body mass index, high C-reactive protein level, depressive symptoms, comorbid ocular diseases, walking disability, self-rated health, and history of congestive heart failure, coronary heart disease, angina, heart attack, stroke, or cancer were reported using means and SEs for continuous variables and numbers and weighted percentages for categorical variables.

We used the unpaired *t* test for the comparison of continuous variables and design-adjusted Rao-Scott Pearson χ^2^ test for the comparison of categorical data to compare the mortality characteristics by AMD status. Plots of survival curves of participants with early, late, and no AMD were generated using Kaplan-Meier estimates. Age- and sex-adjusted Cox proportional hazards regression models were used to estimate hazard ratios (HRs) and 95% CIs for mortality to determine baseline characteristics significantly associated with the end point. Covariates significantly associated with mortality and AMD status were added to final Cox proportional hazards regression models calculating HRs and population-attributable risk of AMD for mortality. Specific-cause mortality risk was estimated after multiple adjustments using the Fine and Gray competing risks regression model.^38^ Mortality resulting from other causes was treated as a competing risk.^38^ To address the nonresponse issue, we used inverse probability weighting to correct the estimates in the sensitivity analyses.^39^ We also conducted sensitivity analyses adjusted for age and age squared in final models to evaluate the nonlinear association of age with mortality. Interactions between covariates were tested, and no evidence of interaction was found (*P* > .05). The proportional hazards assumption for each variable was tested by graphically inspecting or by checking their interaction with follow-up time. No evidence suggested that any of these variables violated the assumption (*P* > .05). The variance inflation factors procedure was used to test collinearity for all variables, and all covariables’ variance inflation factors were less than 2.00 (mean [SE], 1.29 [0.04]). All data analysis was performed using Stata software (version 14.0; StataCorp). Two-sided *P* < .05 was considered significant for statistical inferences.

## Results

Of the total 20 497 participants in 2005-2008 NHANES surveys, 6797 were 40 years or older. Of these, 1194 were excluded owing to missing retinal images (969 participants), ungradable images (224 participants), and missing mortality data (1 participant). The remaining 5603 participants (86.0%; 2810 [47.4%] men and 2793 [52.6%] women; 3017 [77.1%] white; mean [SE] age, 56.4 [0.4] years) were included in the final analytical sample (eFigure in the Supplement). Excluded participants were significantly older (≥80 years, 283 [18.4%] vs 464 [5.2%]; *P* < .001) and more likely to be black (330 [16.2%] vs 1139 [9.6%]; *P* < .001) when compared with study participants. Other demographic, health-related behaviors, and characteristics of excluded and included participants are shown in eTable 1 in the Supplement. Demographic characteristics, health-related behaviors, and general health comorbidities of participants overall and by AMD status are presented in Table 1. Participants with any AMD tended to be older (≥80 years, 143 [27.4%] vs 321 [3.6%]), white (314 [86.5%] vs 2703 [76.4%]), unmarried (206 [40.2%] vs 1819 [30.2%]), and former smokers (178 [42.0%] vs 1634 [30.1%]); to have hypertension (281 [59.8%] vs 2477 [42.5%]) and dyslipidemia (170 [41.3%] vs 1961 [37.6%]); to be normal or overweight (295 [68.6%] vs 3077 [61.5%]); to have walking disability (88 [18.1%] vs 507 [7.5%]); and to have comorbid CVD (eg, stroke, 56 [12.4%] vs 230 [3.5%]) and cancer (81 [16.3%] vs 617 [12.0%]). Other characteristics did not differ between the groups with and without AMD.

**Table 1.** Demographics, Health Behaviors, and General Health Characteristics of Participants With and Without AMD^a^

Characteristic	Study Participants		
	All (N = 5603)	Without AMD (n = 5162)	With AMD (n = 441)
Age, No. (%), y			
40-49	1501 (34.6)	1471 (36.2)	30 (12.0)
50-59	1327 (29.8)	1281 (30.9)	46 (15.1)
60-69	1386 (18.8)	1294 (18.7)	92 (20.5)
70-79	925 (11.6)	795 (10.6)	130 (25.0)
≥80	464 (5.2)	321 (3.6)	143 (27.4)
Sex, No. (%)			
Male	2810 (47.4)	2581 (47.4)	229 (47.0)
Female	2793 (52.6)	2581 (52.6)	212 (53.0)
Race/ethnicity, No. (%)			
Non-Hispanic white	3017 (77.1)	2703 (76.4)	314 (86.5)
Non-Hispanic black	1139 (9.6)	1103 (10.0)	36 (3.8)
Mexican American	864 (5.4)	811 (5.5)	53 (4.2)
Other	583 (7.9)	545 (8.1)	38 (5.5)
Educational attainment, No. (%)			
<High school	1643 (18.0)	1511 (17.7)	132 (22.6)
≥High school	3960 (82.0)	3651 (82.3)	309 (77.4)
Marital status, No. (%)			
Unmarried or other	2025 (30.9)	1819 (30.2)	206 (40.2)
Married or living with a partner	3576 (69.1)	3341 (69.8)	235 (59.8)
Poverty income ratio, No. (%)			
Below poverty line (<1.00)	828 (9.3)	766 (9.3)	62 (10.0)
At or above poverty line (≥1.00)	4380 (90.7)	4043 (90.7)	337 (90.0)
Smoking status, No. (%)			
Never	2648 (48.5)	2451 (48.9)	197 (43.0)
Former	1812 (30.9)	1634 (30.1)	178 (42.0)
Current	1141 (20.6)	1075 (21.0)	66 (15.0)
Alcohol consumption, No. (%)			
Lifetime abstainer or former	1358 (20.6)	1233 (20.2)	125 (26.4)
Current, drinks/wk			
≤3	2961 (55.4)	2741 (55.6)	220 (52.4)
>3	1148 (24.0)	1061 (24.2)	87 (21.2)
Diabetes, No. (%)	1053 (13.6)	973 (13.4)	80 (16.4)
Hypertension, No. (%)	2758 (43.6)	2477 (42.5)	281 (59.8)
High total cholesterol level, No. (%)	2131 (37.9)	1961 (37.6)	170 (41.3)
LDL-C:HDL-C level ratio, mean (SE)	2.30 (0.02)	2.32 (0.02)	2.11 (0.07)
BMI, No. (%)			
<18.5	79 (1.3)	75 (1.4)	4 (0.5)
18.5-30.0	3372 (62.0)	3077 (61.5)	295 (68.6)
≥30.0	2109 (36.7)	1970 (37.1)	139 (30.9)
High C-reactive protein level, No. (%)	627 (10.6)	568 (10.4)	59 (12.8)
Depressive symptoms, No. (%)	463 (7.2)	433 (7.3)	30 (6.7)
Comorbid ocular diseases, No. (%)	1361 (19.3)	1174 (17.8)	187 (40.0)
Walking disability, No. (%)	595 (8.2)	507 (7.5)	88 (18.1)
Self-rated health, No. (%)			
Poor to fair	1427 (18.8)	1305 (18.5)	122 (23.0)
Good to excellent	4056 (81.2)	3746 (81.5)	310 (77.0)
History of congestive heart failure, No. (%)	256 (3.3)	223 (3.0)	33 (7.3)
History of coronary heart disease, No. (%)	319 (4.8)	275 (4.4)	44 (10.1)
History of angina, No. (%)	230 (3.4)	200 (3.2)	30 (6.0)
History of heart attack, No. (%)	350 (4.9)	299 (4.5)	51 (10.1)
History of stroke, No. (%)	286 (4.1)	230 (3.5)	56 (12.4)
History of cancer, No. (%)	698 (12.2)	617 (12.0)	81 (16.3)

Abbreviations: AMD, age-related macular degeneration; BMI, body mass index (calculated as weight in kilograms divided by height in meters squared); HDL-C, high-density lipoprotein cholesterol; LDL-C, low-density lipoprotein cholesterol.

^a^ All proportions, means, and SEs are weighted estimates of the US population characteristics, taking into account the complex sampling design of the National Health and Nutrition Examination Survey.

### All-Cause Mortality

Among the 5603 participants included in the current analysis, AMD was present at the baseline examination in 441 participants (6.6%), of whom 386 (5.8%) had early and 55 (0.8%) had late AMD. After a median follow-up of 4.5 years (interquartile range, 3.6-5.6 years), 433 participants (5.3%) died of all causes. Among these deceased participants, 361 (83.1%) had no AMD at baseline; 54 (11.5%), signs of early AMD; 18 (5.4%), signs of late AMD; and 72 (16.9%), any AMD. Mortality rates were higher for those participants who had early (54 [10.6%]), late (18 [35.9%]), or any (72 [13.6%]) AMD compared with no AMD (361 [4.7%]) (Table 2). The mean (SE) age at death of participants without AMD (70.9 [1.0] years) was significantly younger than that of participants with early (80.4 [1.4] years; *P* < .001), late (83.1 [1.7] years; *P* < .001), or any (81.3 [1.1] years; *P* < .001) AMD. The mean time to death did not differ significantly by AMD status.

**Table 2.** Mortality Characteristics Overall and by AMD Status^a^

Characteristics	All Participants (N = 5603)	AMD Status^b^			
		None (n = 5162)	Any (n = 441)	Early (n = 386)	Late (n = 55)
Age at death, mean (SE), y					
Due to all causes	72.6 (0.9)	70.9 (1.0)	81.3 (1.1)^c^	80.4 (1.4)^c^	83.1 (1.7)^c^
Due to CVD	73.8 (1.3)	72.8 (1.4)	79.1 (3.0)	77.9 (3.5)	83.7 (3.5)^d^
Due to cancer	70.9 (1.1)	70.0 (1.3)	78.4 (2.9)^d^	77.1 (3.3)	84.8 (1.9)^c^
Due to non-CVD and noncancer causes	72.9 (1.3)	70.3 (1.3)	82.9 (1.1)^c^	83.0 (1.4)^c^	82.8 (1.6)^c^
Mortality rate, No. (%)					
Due to all causes	433 (5.3)	361 (4.7)	72 (13.6)^c^	54 (10.6)^d^	18 (35.9)^d^
Due to CVD	117 (1.4)	102 (1.2)	15 (3.3)^c^	12 (3.0)^d^	3 (5.4)^d^
Due to cancer	105 (1.3)	92 (1.3)	13 (2.2)^c^	11 (2.1)^d^	2 (2.9)^d^
Due to non-CVD and noncancer causes	211 (2.6)	167 (2.2)	44 (8.2)^c^	31 (5.5)^d^	13 (27.6)^d^
Time to death from baseline examination, mean (SE), mo					
Due to all causes	32.8 (1.0)	32.4 (1.2)	34.6 (2.1)	34.0 (2.5)	35.9 (5.4)
Due to CVD	31.1 (1.5)	30.0 (1.8)	36.8 (4.9)	30.0 (3.5)	64.4 (4.4)^c^
Due to cancer	35.6 (2.0)	36.0 (2.1)	32.2 (2.8)	32.2 (3.3)	32.0 (2.4)
Due to non-CVD and noncancer causes	32.2 (1.3)	31.7 (1.7)	34.4 (2.8)	36.9 (3.9)	30.8 (4.8)

Abbreviations: AMD, age-related macular degeneration; CVD, cardiovascular disease.

^a^ Mortality was assessed through December 31, 2011. All proportions, means, and SEs are weighted estimates of the US population characteristics, taking into account the complex sampling design of the National Health and Nutrition Examination Survey.

^b^ All *P* values were calculated using the unpaired *t* test for continuous variables and the design-adjusted Rao-Scott Pearson χ^2^ test for categorical variables. Comparisons were between each group with AMD and the group with no AMD and were unadjusted.

^c^ *P* < .001.

^d^ *P* < .05.

The association of baseline covariates with all-cause mortality is shown in Table 3. After adjustments for age and sex, the HRs increased exponentially for each decade of age. Men had an increased risk of mortality due to all causes (HR, 1.53; 95% CI, 1.20-1.96; *P* = .001). Age- and sex-adjusted Cox proportional hazards regression models showed covariates including race/ethnicity (HR, 1.67; 95% CI, 1.25-2.22), educational attainment (HR, 0.61; 95% CI, 0.46-0.81), marital status (HR, 0.51; 95% CI, 0.41-0.64), family income (HR, 0.43; 95% CI, 0.30-0.62), smoking status (HR for former smokers, 1.63 [95% CI, 1.12-2.36]; HR for current smoking, 3.54 [95% CI, 2.57-4.87]), alcohol consumption (HR, 0.51; 95% CI, 0.38-0.70), diabetes (HR, 2.25; 95% CI, 1.52-3.31), dyslipidemia (HR, 0.72; 95% CI, 0.58-0.91), body mass index (HR, 3.29; 95% CI, 1.61-6.75), C-reactive protein level (HR, 2.62; 95% CI, 1.64-4.20), depressive symptoms (HR, 2.16; 95% CI, 1.37-3.42), comorbid ocular diseases (HR, 2.01; 95% CI, 1.49-2.72), self-rated health status (HR, 0.36; 95% CI, 0.28-0.47), walking disability (HR, 2.99; 95% CI, 2.36-3.78), and self-reported history of CVD (eg, HR for stroke, 2.63; 95% CI, 1.98-3.50) or cancer (HR, 1.50; 95% CI, 1.13-2.01) were significantly associated with an increased risk of all-cause mortality. After controlling for variables significantly associated with mortality and AMD status, the multivariate Cox regression model (Table 4) indicated that poorer survival was associated with late AMD at baseline when compared with participants without AMD (HR, 2.01; 95% CI, 1.00-4.03; *P* = .049). The stratum-specific HRs increased exponentially for each decade of age, ranging from 2.59 (95% CI, 1.36-4.94) for the group aged 40 to 49 years and 19.4 (95% CI, 9.18-41.0) for the group 80 years or older. However, participants with early AMD (HR, 0.79; 95% CI, 0.57-1.11; *P* = .17) or any AMD (HR, 1.00; 95% CI, 0.75-1.33; *P* = .97) at baseline were not at greater risk of all-cause mortality compared with participants without AMD. The population-attributable risk ranged from −1.23% (95% CI, −2.56% to 0.63%) for early AMD to 0.80% (95% CI, 0.00%-2.37%) for late AMD (Table 4). Multiple adjusted Kaplan-Meier curves for all-cause mortality by AMD status are shown in the Figure.

**Table 3.** All-Cause Mortality by Demographic, Health-Related Behaviors, and General Health Characteristics^a^

Characteristics	Participants		HR (95% CI)^b^
	Survived (n = 5170)	Died (n = 433)	
Age, No. (%), y			
40-49	1473 (36.0)	28 (8.8)	1 [Reference]
50-59	1281 (30.7)	46 (15.0)	2.03 (1.13-3.64)^c^
60-69	1298 (18.9)	88 (16.5)	3.67 (1.89-7.12)^d^
70-79	803 (10.6)	122 (28.7)	10.9 (5.64-21.2)^d^
≥80	315 (3.8)	149 (31.0)	30.4 (16.5-55.8)^d^
Sex, No. (%)			
Male	2548 (47.0)	262 (54.0)	1.53 (1.20-1.96)^c^
Female	2622 (53.0)	171 (46.0)	1 [Reference]
Race/ethnicity, No. (%)			
Non-Hispanic white	2742 (77.0)	275 (79.2)	1 [Reference]
Non-Hispanic black	1042 (9.4)	97 (11.9)	1.67 (1.25-2.22)^c^
Mexican American	827 (5.5)	37 (3.7)	1.00 (0.70-1.42)
Other	559 (8.1)	24 (5.2)	0.97 (0.47-2.01)
Educational attainment, No. (%)			
Less than high school	1462 (17.1)	181 (33.6)	1 [Reference]
High school or more	3708 (82.9)	252 (66.4)	0.61 (0.46-0.81)^c^
Marital status, No. (%)			
Unmarried or other	1800 (29.7)	225 (51.6)	1 [Reference]
Married or living with a partner	3368 (70.3)	208 (48.4)	0.51 (0.41-0.64)^d^
Poverty income ratio, No. (%)			
Below poverty line (<1.00)	737 (8.9)	91 (17.5)	1 [Reference]
At or above poverty line (≥1.00)	4078 (91.1)	302 (82.5)	0.43 (0.30-0.62)^d^
Smoking status, No. (%)			
Never	2502 (49.4)	146 (31.5)	1 [Reference]
Former	1624 (30.2)	188 (43.6)	1.63 (1.12-2.36)^c^
Current	1042 (20.4)	99 (24.9)	3.54 (2.57-4.87)^d^
Alcohol consumption, No. (%)			
Lifetime abstainer or former	1222 (20.0)	136 (31.0)	1 [Reference]
Current, drinks/wk			
≤3	2731 (55.4)	230 (54.9)	0.87 (0.70-1.07)
>3	1092 (24.6)	56 (14.1)	0.51 (0.38-0.70)^d^
Diabetes, No. (%)			
No	4122 (87.2)	289 (70.5)	1 [Reference]
Yes	924 (12.8)	129 (29.5)	2.25 (1.52-3.31)^d^
Hypertension, No. (%)			
No	2617 (57.6)	133 (33.9)	1 [Reference]
Yes	2479 (42.4)	279 (66.1)	1.32 (0.94-1.83)
High cholesterol level, No. (%)			
No	3064 (62.1)	258 (62.5)	1 [Reference]
Yes	1979 (37.9)	152 (37.5)	0.72 (0.58-0.91)^c^
LDL-C:HDL-C level ratio, mean (SE)	2.31 (0.02)	2.19 (0.10)	1.07 (0.80-1.42)
BMI, No. (%)			
18.5-30.0	3081 (61.8)	291 (65.6)	1 [Reference]
<18.5	66 (1.2)	13 (3.7)	3.29 (1.61-6.75)^c^
≥30.0	1988 (37.0)	121 (30.8)	1.03 (0.79-1.34)
High C-reactive protein level, No. (%)			
No	4472 (90.0)	318 (78.1)	1 [Reference]
Yes	539 (10.0)	88 (21.9)	2.62 (1.64-4.20)^d^
Depressive symptoms, No. (%)			
No	4613 (92.9)	372 (90.6)	1 [Reference]
Yes	422 (7.1)	41 (9.4)	2.16 (1.37-3.42)^c^
Comorbid ocular diseases, No. (%)			
No	3895 (82.5)	190 (47.7)	1 [Reference]
Yes	1139 (17.5)	222 (52.3)	2.01 (1.49-2.72)^d^
Walking disability, No. (%)			
No	4703 (93.0)	305 (70.4)	1 [Reference]
Yes	467 (7.0)	128 (29.6)	2.99 (2.36-3.78)^d^
Self-rated health, No. (%)			
Poor to fair	1238 (17.5)	189 (41.5)	1 [Reference]
Good to excellent	3822 (82.5)	234 (58.5)	0.36 (0.28-0.47)^d^
History of congestive heart failure, No. (%)			
No	4993 (97.6)	354 (79.9)	1 [Reference]
Yes	177 (2.4)	79 (20.1)	4.23 (3.02-5.91)^d^
History of coronary heart disease, No. (%)			
No	4900 (95.5)	384 (89.1)	1 [Reference]
Yes	270 (4.5)	49 (10.9)	1.08 (0.75-1.57)
History of angina, No. (%)			
No	4975 (96.9)	398 (92.2)	1 [Reference]
Yes	195 (3.1)	35 (7.8)	1.18 (0.82-1.71)
History of heart attack, No. (%)			
No	4898 (95.8)	355 (83.1)	1 [Reference]
Yes	272 (4.2)	78 (16.9)	2.02 (1.32-3.10)^c^
History of stroke, No. (%)			
No	4952 (96.6)	365 (83.3)	1 [Reference]
Yes	218 (3.4)	68 (16.7)	2.63 (1.98-3.50)^d^
History of cancer, No. (%)			
No	4583 (88.6)	322 (72.4)	1 [Reference]
Yes	587 (11.4)	111 (27.6)	1.50 (1.13-2.01)^c^

Abbreviations: AMD, age-related macular degeneration; BMI, body mass index (calculated as weight in kilograms divided by height in meters squared); HDL-C, high-density lipoprotein cholesterol; HR, hazard ratio; LDL-C, low-density lipoprotein cholesterol.

^a^ All-cause mortality was assessed through December 31, 2011. All proportions, means, and SEs are weighted estimates of the US population characteristics, taking into account the complex sampling design of the National Health and Nutrition Examination Survey.

^b^ Adjusted for age and sex.

^c^ *P* < .05.

^d^ *P* < .001.

**Table 4.** Cox Proportional Hazards Models for All-Cause Mortality and Fine and Gray Competing Risks Regression Models for Specific-Cause Mortality by AMD Status

AMD Status	Mortality^a^							
	All-Cause		CVD-Specific		Cancer-Specific		Not Due to CVD or Cancer	
	HR (95% CI)	PAR (95% CI), %	HR (95% CI)	PAR (95% CI), %	HR (95% CI)	PAR (95% CI), %	HR (95% CI)	PAR (95% CI), %
None	1 [Reference]	1 [Reference]	1 [Reference]	1 [Reference]	1 [Reference]	1 [Reference]	1 [Reference]	1 [Reference]
Any (early or late)	1.00 (0.75 to 1.33)	0.00 (−1.68 to 2.13)	0.55 (0.20 to 1.50)	−3.06 (−5.57 to 3.21)	0.88 (0.43 to 1.82)	−0.77 (−3.91 to 5.12)	1.33 (0.86 to 2.07)	2.14 (−0.96 to 6.60)
Early	0.79 (0.57 to 1.11)	−1.23 (−2.56 to 0.63)	0.47 (0.17 to 1.29)	−3.20 (−5.07 to 1.65)	0.85 (0.40 to 1.78)	−0.89 (−3.59 to 4.35)	0.96 (0.58 to 1.59)	−0.25 (−2.52 to 3.31)
Late	2.01 (1.00 to 4.03)^b^	0.80 (0.00 to 2.37)^b^	0.78 (0.14 to 4.35)	−0.17 (−0.69 to 2.61)	1.27 (0.19 to 8.68)	0.21 (−0.66 to 5.79)	3.42 (1.38 to 8.49)^b^	1.90 (0.30 to 5.65)^b^

Abbreviations: AMD, age-related macular degeneration; CVD, cardiovascular disease; HR, hazard ratio; PAR, population attributable risk.

^a^ Adjusted for age, sex, race/ethnicity, educational attainment, marital status, family income, smoking status, alcohol consumption, diabetes, hypertension, high cholesterol level, body mass index, high C-reactive protein level, depressive symptoms, comorbid ocular diseases, walking disability, self-rated health, history of CVD, and cancer.

^b^ *P* < .05.

**Figure.**
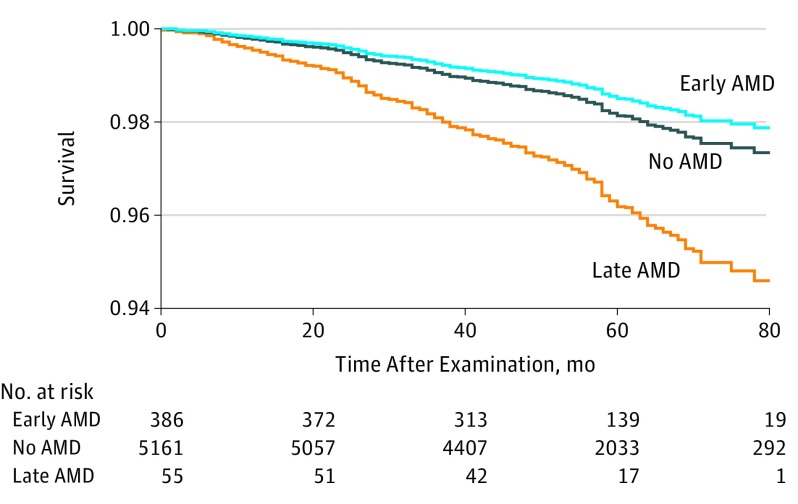
Adjusted Kaplan-Meier Curve for All-Cause Mortality Rate Findings are stratified by age-related macular degeneration (AMD) status, using the 2005-2008 National Health and Nutrition Examination Survey data. All-cause mortality was assessed through December 31, 2011. Late AMD was associated with greater mortality rates.

### Cause-Specific Mortality

Among the 433 participants who died of all causes, 117 deaths (25.6%) were CVD specific, 105 (25.2%) were cancer specific, and 211 (49.2%) were not specific to CVD or cancer. The presence of any, early, or late AMD was associated with significantly higher mortality rates for each specific cause (Table 2). Competing risk regression models for cause-specific mortality showed that late AMD was associated with a more than 3-fold higher risk of mortality not due to CVD or cancer (HR, 3.42; 95% CI, 1.38-8.49; *P* = .01) after multiple adjustments. However, we identified no association of any AMD or early AMD with specific-cause mortality after multivariate adjustments. The population-attributable risk results of these risk factors are listed in Table 4.

### Sensitivity Analyses

The sensitivity analyses used inverse probability weighting to correct the estimates for nonresponse, which yielded results similar to those reported in the main analysis (eTable 2 in the Supplement). We also observed results comparable to those of the main analysis when squared age was included in the final model (eTable 3 in the Supplement).

## Discussion

In this nationally representative sample consisting of 5603 US adults 40 years or older, we report that only late AMD was associated with increased risks of all-cause mortality and mortality not specific to CVD or cancer. The presence of any AMD and early AMD were not associated with all-cause or specific-cause mortality.

The findings from previous population-based studies^12,13,14,15,16,17,18,19,20,21,22,23,24,25,40,41^ on associations between AMD and mortality are summarized in eTable 4 in the Supplement. Our finding that the presence of late AMD was associated with an increased risk of all-cause mortality is in line with the Age-Related Eye Disease Study (AREDS),^14^ AREDS2,^15^ and Beaver Dam Eye Study.^16,20^ Other population-based studies^12,13,17,18,19^ have reported significant associations between any and mild AMD and all-cause mortality in subpopulations only. In contrast, the Singapore Malay Eye study,^22^ Beijing Eye study,^24^ Rotterdam study,^21^ Melbourne Visual Impairment Project cohort,^25^ Andhra Pradesh Eye Disease study,^40^ a UK study,^23^ and the Atherosclerosis Risk in Communities Study^41^ did not report a significant association between AMD and survival. The differences in definitions and numbers of confounders, the assessment and grading system of AMD, the length of the follow-up period, and the number of deaths may explain varied results from these studies.

In the analysis of cause-specific mortality, our results support those of a recent meta-analysis of 5 population-based studies^26^ that suggest that AMD is not associated with CVD mortality. However, our findings were challenged by 2 recent meta-analyses^42,43^ that concluded that AMD, specifically late AMD, was associated with an increased risk of CVD mortality. The inconsistent nature of these results may be attributable to a range of factors. Given that atherothrombotic events and mortality have been associated with the use of anti-VEGF agents in patients with late AMD,^27,28^ undocumented use of anti-VEGF therapies may overestimate poor survival resulting from CVD in patients with AMD. In addition, when estimating the specific mortality in a geriatric population with comorbidities associated with poorer survival, the use of Cox proportional hazards regression models in previous studies might lead to overestimation in the absolute risk of the specific mortality by not considering the competing risks of death.^38^ We used the competing risk models to deal with this methodologic issue. Last, some previous studies were subject to insufficient adjustment for important confounders, such as smoking^44^ and a history of CVD events,^14^ that could explain the significant association between AMD and CVD mortality.

Our association between late AMD and mortality not specific to CVD or cancer is in agreement with several previous studies,^15,45^ but not others.^12^ The reasons underlying the association of AMD with increased risk of mortality not due to CVD or cancer are still unclear. However, growing evidence supports the association between AMD and neurodegenerative diseases (eg, Alzheimer disease),^46^ which increased the risk of mortality.^47^ The limited number of deaths due to Alzheimer disease in our analysis could not explore this hypothesis. In addition, it has been speculated that visual impairment and blindness due to AMD may lead to functional and psychological problems, such as falls,^48,49^ fractures,^50,51^ unintentional injuries,^52^ and a loss of independence.^53,54,55^ These problems may contribute to the higher risk of mortality not due to CVD or cancer when compared with unaffected individuals. However, this hypothesis has been challenged by previous analyses.^14,15^ We did not have sufficient unintentional injury–related mortality to examine this hypothesis. We found no statistically significant differences in depression symptoms between the groups with any AMD and no AMD. Although depression symptoms were associated with higher risk of mortality in the age- and sex-adjusted model, it did not remain significant after multiple adjustments. Further studies are needed to elucidate these associations.

Another explanation elucidating the poor survival among individuals with late AMD is that the AMD reflects systemic comorbidities associated with frailty and aging. This explanation is supported by results from the present study and previous studies.^7,14,26,56^ Common pathogenesis, such as chronic inflammation, atherosclerosis, oxidative stress, and lipid metabolism might be the main mechanism between AMD and systemic comorbidities.^56,57,58^ The implications of these findings suggest that AMD is a biomarker of frailty and aging.^59,60^ Alternatively, this association between AMD and mortality may be attributable to unmeasured or inadequately assessed confounding factors for AMD. Age is the most important risk factor for AMD and mortality. Inclusion of age assessed as a continuous or a categorical variable or additional inclusion of age squared in the final model did not affect the association between late AMD and mortality. Second to age, smoking is an established risk factor for AMD.^4^ In the models adjusted for multiple covariates, AMD remained significantly associated with mortality. In addition, interaction terms of AMD with age or smoking status were not significantly associated with mortality, suggesting no difference in the AMD-mortality association in these subgroups. Extensive evidence supported increased risks of mortality among participants with other age-related ocular diseases.^61,62,63^ To elaborate on the real nature of the AMD-mortality association, we also adjusted for comorbid ocular diseases in the final model, which did not affect the AMD-mortality association.

### Strengths and Limitations

Strengths of these analyses include the large sample size of an elderly cohort, standardized objective methods for assessing AMD, availability of comprehensive demographic characteristics, health indicators, comorbidities, and complete death records. The study was limited by the following points. First, health behavior and comorbidities were collected at a single time point, and study participants’ behavior and comorbidity status might change during follow-up. Second, although we adjusted for a comprehensive range of confounding factors, we cannot rule out residual confounding, such as anti-VEGF therapies. Finally, participants excluded in the present analysis were older and unhealthier, which might have influenced results. Nevertheless, the inverse probability weighting model to correct the estimates for nonresponse yielded similar results, again verifying the robustness of our conclusions.

## Conclusions

Our findings suggest that in a large sample of elderly participants residing in the United States, only late AMD was associated with an increased risk of all-cause morality and mortality not related to CVD or cancer. The reasons for the association of late AMD with decreased survival have yet to be confirmed; however, our results suggest that AMD may reflect some systemic pathologic comorbidities indicative of frailty and aging. Alternatively, this association may be due to unmeasured or inadequately assessed confounding factors for late AMD. Further studies are needed to confirm these findings and elucidate the possible mechanisms underlying AMD.

## Notes

Supplement. **eFigure.** Schematic Showing Inclusion Criteria for Study Participants **eTable 1**. Demographic, Health-Related Behaviors and General Health Characteristics of Participants Included and Excluded in the Analyses **eTable 2**. Cox Proportional Hazards Models for All-Cause Mortality and Fine and Gray Competing Risks Regression Models for Specific-Cause Mortality by Age-Related Macular Degeneration Status Using Inverse Probability Weighting **eTable 3**. Cox Proportional Hazards Models for All-Cause Mortality and Fine and Gray Competing Risks Regression Models for Specific-Cause Mortality by Age-Related Macular Degeneration Status With Additional Adjustment for Age Squared **eTable 4**. Summary Description of Previous Studies on the Association Between Age-Related Macular Degeneration and Mortality
