# Supplementary material for: Association of Age-Related Macular Degeneration With Risk of All-Cause and Specific-Cause Mortality in the National Health and Nutrition Examination Survey, 2005 to 2008
Source: JAMA Ophthalmol. 2018 Dec 20;137(3):248–57. doi: 10.1001/jamaophthalmol.2018.6150 (PMC6439709; doi:10.1001/jamaophthalmol.2018.6150)
Supplement: Supplement. — eFigure. Schematic Showing Inclusion Criteria for Study Participants eTable 1. Demographic, Health-Related Behaviors and General Health Characteristics of Participants Included and Excluded in the Analyses eTable 2. Cox Proportional Hazards Models for All-Cause Mortality and Fine and Gray Competing Risks Regression Models for Specific-Cause Mortality by Age-Related Macular Degeneration Status Using Inverse Probability Weighting eTable 3. Cox Proportional Hazards Models for All-Cause Mortality and Fine and Gray Competing Risks Regression Models for Specific-Cause Mortality by Age-Related Macular Degeneration Status With Additional Adjustment for Age Squared eTable 4. Summary Description of Previous Studies on the Association Between Age-Related Macular Degeneration and Mortality [file jamaophthalmol-137-248-s001.pdf]

Supplementary Online Content

Zhu Z, Wang W, Keel S, Zhang J, He M. Association of age-related macular degeneration with risk of all-cause and specific-cause mortality in the National Health and Nutrition Examination Survey, 2005 to 2008. *JAMA Ophthalmol*. Published online December 20, 2018. doi:10.1001/jamaophthalmol.2018.6150

eFigure. Schematic Showing Inclusion Criteria for Study Participants

eTable 1. Demographic, Health-Related Behaviors and General Health Characteristics of Participants Included and Excluded in the Analyses

eTable 2. Cox Proportional Hazards Models for All-Cause Mortality and Fine and Gray Competing Risks Regression Models for Specific-Cause Mortality by Age-Related Macular Degeneration Status Using Inverse Probability Weighting

eTable 3. Cox Proportional Hazards Models for All-Cause Mortality and Fine and Gray Competing Risks Regression Models for Specific-Cause Mortality by Age-Related Macular Degeneration Status With Additional Adjustment for Age Squared

eTable 4. Summary Description of Previous Studies on the Association Between Age-Related Macular Degeneration and Mortality

This supplementary material has been provided by the authors to give readers additional information about their work.

© 2018 Zhu Z et al. *JAMA Ophthalmology.*

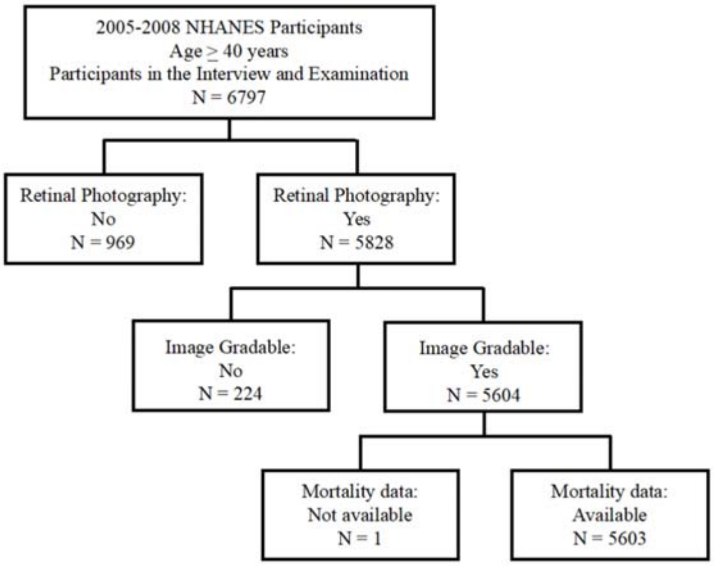

eFigure. Schematic Showing Inclusion Criteria for Study Participants

Schematic showing study participants included for the present analysis from the 2005-2008 National Health and Nutrition Examination Survey(NHANES). A total of 5603 participants were included.

© 2018 Zhu Z et al. *JAMA Ophthalmology.*

eTable 1. Demographic, Health-Related Behaviors and General Health Characteristics of Participants Included and Excluded in the Analyses [a]

**Characteristic**	**No. of Excluded Subjects (n = 1194)**	**No. of Included Subjects (n = 5603)**	***P* Value** [b]
Age, No. (%)			
40-49	241 (27.4)	1501 (34.6)	**<.001**
50-59	195 (23.5)	1327 (29.8)	
60-69	238 (15.5)	1386 (18.8)	
70-79	237 (15.2)	925 (11.6)	
≥80	283 (18.4)	464 (5.2)	
Sex, No. (%)			
Male	563 (45.2)	2810 (47.4)	.23
Female	631 (54.8)	2793 (52.6)	
Race/ethnicity, No. (%)			
Non-Hispanic white	529 (66.4)	3017 (77.1)	**<.001**
Non-Hispanic black	330 (16.2)	1139 (9.6)	
Mexican American	184 (6.2)	864 (5.4)	
Other	151 (11.2)	583 (7.9)	
Educational attainment, No. (%)			
<high school	498 (28.8)	1643 (18.0)	**<.001**
≥High school	696 (71.2)	3960 (82.0)	
Marital status, No. (%)			
Unmarried or other	551 (39.3)	2025 (30.9)	**<.001**
Married or living with a partner	639 (60.7)	3576 (69.1)	
Poverty income ratio, No. (%)			
Below poverty line (<1.00)	216 (13.3)	828 (9.3)	**.01**
At or above poverty line (≥1.00)	844 (86.7)	4380 (90.7)	

© 2018 Zhu Z et al. *JAMA Ophthalmology.*

Smoking status, No. (%)			
Never	647 (54.3)	2648 (48.5)	.06
Former	345 (27.8)	1812 (30.9)	
Current	197 (17.9)	1141 (20.6)	
Alcohol consumption, No. (%)			
Lifetime abstainer or former	277 (32.3)	1358 (20.6)	**<.001**
Current, drinks/wk			
≤3	372 (49.5)	2961 (55.4)	
>3	126 (18.2)	1148 (24.0)	
Diabetes, No. (%)	343 (25.8)	1053 (13.6)	**<.001**
Hypertension, No. (%)	581 (54.4)	2758 (43.6)	**<.001**
High total cholesterol level, No. (%)	431 (40.0)	2131 (37.9)	.33
LDL-C:HDL-C level ratio, mean (SE)	2.20 ± 0.04	2.30 ± 0.02	**.02**
BMI, No. (%)			
< 18.5	24 (2.4)	79 (1.3)	.17
18.5-30.0	658 (62.2)	3372 (62.0)	
≥30.0	396 (35.4)	2109 (36.7)	
High C-reactive protein level, No. (%)	130 (11.8)	627 (10.6)	.34
Depressive symptoms, No. (%)	61 (7.2)	463 (7.2)	.99
Comorbid ocular diseases, No. (%)	319 (88.4)	1361 (19.3)	**<.001**
Walking disability, No. (%)	307 (21.5)	595 (8.2)	**<.001**
Self-rated health, No. (%)			
Poor to fair	303 (30.1)	1427 (18.8)	**<.001**
Good to excellent	483 (69.9)	4056 (81.2)	
History of congestive heart failure, No. (%)	105 (6.8)	256 (3.3)	**<.001**
History of coronary heart disease, No. (%)	96 (7.2)	319 (4.8)	**.007**

© 2018 Zhu Z et al. *JAMA Ophthalmology.*

History of angina, No. (%)	67 (4.8)	230 (3.4)	.07
History of heart attack, No. (%)	109 (7.6)	350 (4.9)	**.002**
History of stroke, No. (%)	124 (7.8)	286 (4.1)	**<.001**
History of cancer, No. (%)	183 (14.9)	698 (12.2)	.07

Abbreviations: AMD, age-related macular degeneration; BMI, body mass index (calculated as weight in kilograms divided by height in meters squared); LDL-C, low-density lipoprotein cholesterol; HDL-C, high-density lipoprotein cholesterol.

[a]All proportions, means, and SEs are weighted estimates of the US population characteristics, taking into account the complex sampling design of the National Health and Nutrition Examination Survey.

[b]All P values were calculated using the unpaired *t* test for continuous variables and the design-adjusted Rao-Scott Pearson χ^2 test for categorical variables. Boldface indicates statistical significance.

© 2018 Zhu Z et al. *JAMA Ophthalmology.*

eTable 2. Cox Proportional Hazards Models for All-Cause Mortality and Fine and Gray Competing Risks Regression Models for Specific-Cause Mortality by Age-Related Macular Degeneration Status Using Inverse Probability Weighting

AMD Status	Mortality [a]							
	All-Cause		CVD-Specific		Cancer-Specific		Not Due to CVD or Cancer	
	HR (95% CI)	PAR (95% CI), %	HR (95% CI)	PAR (95% CI), %	HR (95% CI)	PAR (95% CI), %	HR (95% CI)	PAR (95% CI), %
None	1 [Reference]	1 [Reference]	1 [Reference]	1 [Reference]	1 [Reference]	1 [Reference]	1 [Reference]	1 [Reference]
Any (early or late)	0.99 (0.76 to 1.29)	-0.07 (-1.61 to 1.88)	0.48 (0.17 to 1.37)	-3.55 (-5.81 to 2.41)	0.90 (0.44 to 1.84)	-0.69 (-3.87 to 5.25)	1.39 (0.92 to 2.11)	2.53 (-0.54 to 6.84)
Early	0.78 (0.56 to 1.07)	-1.29 (-2.62 to 0.40)	0.40 (0.13 to 1.20)	-3.61 (-5.30 to 1.15)	0.84 (0.40 to 1.77)	-0.91 (-3.58 to 4.25)	1.00 (0.61 to 1.65)	0.01 (-2.33 to 3.64)
Late	1.96 (1.06 to 3.62)[b]	0.76 (0.05 to 2.05)[b]	0.70 (0.12 to 3.99)	-0.24 (-0.71 to 2.33)	1.33 (0.19 to 9.46)	0.27 (-0.65 to 6.34)	3.27 (1.43 to 7.47)[b]	1.79 (0.35 to 4.92)[b]

Abbreviations: AMD, age-related macular degeneration; CVD, cardiovascular disease; HR, hazard ratio; PAR, population attributable risk.

[a]Adjusted for age, sex, race/ethnicity, educational attainment, marital status, family income, smoking status, alcohol consumption, diabetes, hypertension, high cholesterol level, body mass index, high C-reactive protein level, depressive symptoms, comorbid ocular diseases, walking disability, self-rated health, history of CVD, and cancer.

[b]$P < .05$

© 2018 Zhu Z et al. *JAMA Ophthalmology.*

eTable 3. Cox Proportional Hazards Models for All-Cause Mortality and Fine and Gray Competing Risks Regression Models for Specific-Cause Mortality by Age-Related Macular Degeneration Status with Additional Adjustment for Age Squared

AMD Status	Mortality [a]							
	All-Cause		CVD-Specific		Cancer-Specific		Not Due to CVD or Cancer	
	HR (95% CI)	PAR (95% CI), %	HR (95% CI)	PAR (95% CI), %	HR (95% CI)	PAR (95% CI), %	HR (95% CI)	PAR (95% CI), %
None	1 [Reference]	1 [Reference]	1 [Reference]	1 [Reference]	1 [Reference]	1 [Reference]	1 [Reference]	1 [Reference]
Any (early or late)	0.95 (0.71 to 1.26)	-0.33 (-1.95 to 1.69)	0.49 (0.17 to 1.43)	-3.46 (-5.80 to 2.78)	0.87 (0.43 to 1.76)	-0.85 (-3.91 to 4.81)	1.29 (0.83 to 2.01)	1.89 (-1.14 to 6.25)
Early	0.75 (0.54 to 1.04)	-1.47 (-2.74 to 0.23)	0.42 (0.14 to 1.24)	-3.51 (-5.26 to 1.38)	0.84 (0.40 to 1.74)	-0.97 (-3.60 to 4.10)	0.93 (0.56 to 1.55)	-0.39 (-2.61 to 3.11)
Late	1.89 (1.00 to 3.59)[b]	0.71 (0.00 to 2.03)[b]	0.66 (0.12 to 3.68)	-0.27 (-0.71 to 2.10)	1.25 (0.19 to 8.36)	0.20 (-0.65 to 5.56)	3.17 (1.36 to 7.40)[b]	1.71 (0.28 to 4.87)[b]

Abbreviations: AMD, age-related macular degeneration; CVD, cardiovascular disease; HR, hazard ratio; PAR, population attributable risk.

[a]Adjusted for age, squared age, sex, race/ethnicity, educational attainment, marital status, family income, smoking status, alcohol consumption, diabetes, hypertension, high cholesterol level, body mass index, high C-reactive protein level, depressive symptoms, comorbid ocular diseases, walking disability, self-rated health, history of CVD, and cancer .

[b]$P < .05$

© 2018 Zhu Z et al. *JAMA Ophthalmology.*

eTable 4. Summary Description of Previous Studies on the Association Between Age-Related Macular Degeneration and Mortality

Study	Country	Age range	Sample size	Median follow-up	HR (95% CI)	AMD assessment	Adjustment for covariates
AREDS 2004 [14]	USA	55-81	4753	6.5	All-cause:<br>Category 2 vs 1:1.09 (0.81-1.46)<br>Category 3 vs 1:1.10 (0.85-1.43)<br>Category 4 vs 1:**1.41 (1.08-1.86)**	Wisconsin Age-Related Maculopathy Grading System	Age, sex, race, education, smoking status, body mass index, diabetes mellitus, angina, cancer, and hypertension.
					CVD-cause:<br>Category 2 vs 1:1.41 (0.83-2.39)<br>Category 3 vs 1:1.45 (0.90-2.33)<br>Category 4 vs 1:**1.92 (1.18-3.12)**		
AREDS2 2017 [15]	USA	50-80	4203	5	All-cause:<br>GA vs Intermediate:1.31 (0.81-2.10)<br>Neovascular vs Intermediate: **1.56 (1.21-2.01)**<br>Simple Scale Score 3 vs 1,2: **1.60 (1.07-2.40)**	Wisconsin Age-Related Maculopathy Grading System	Age, gender, race, education, smoking status, diabetes, history of high blood pressure, history of congestive heart failure, history of coronary heart disease, history of myocardial infarction, history of stroke, and antiangiogenic treatment.

© 2018 Zhu Z et al. *JAMA Ophthalmology.*

					Simple Scale Score 4 vs 1,2: **1.72 (1.19-2.50)**		
					CVD-cause:<br>GA vs Intermediate:1.35 (0.57-3.17)<br>Neovascular vs Intermediate: **1.68 (1.04-2.71)**<br>Simple Scale Score 3 vs 1,2:1.45 (0.69-3.03)<br>Simple Scale Score 4 vs 1,2:1.56 (0.79-3.07)		
AGES 2014 [13]	Iceland	67-96	4910	8.6	≥ 83 yrs<br>All-cause:<br>Early vs no AMD:1.21 (0.90-1.62)<br>Late vs no AMD:**1.76 (1.20-2.57)**	Wisconsin Age-Related Maculopathy Grading System	Age, established mortality risk factors, and comorbid conditions.
					CVD-cause:<br>Early vs no AMD:1.42 (0.94-2.12)<br>Late vs no AMD:**2.37 (1.41, 3.98)**		

© 2018 Zhu Z et al. *JAMA Ophthalmology.*

SOF 2015 [12]	USA	≥ 65	1202 women	9.5	All-cause:<br>Early vs no AMD:1.08 (0.91-1.28)<br>Late vs no AMD:1.04 (0.65-1.68)<br>Any vs no AMD:1.10 (0.93-1.30)<br>CVD-cause:<br>Early vs no AMD:1.09 (0.82-1.46)<br>Late vs no AMD:1.66 (0.87-3.18)<br>Any vs no AMD:1.18 (0.89-1.56)	Wisconsin Age-Related Maculopathy Grading System	Age, race, self-reported frailty, body mass index (BMI), Mini-Mental State Examination (MMSE) score, walking speed, history of congestive heart failure (CHF), history of myocardial infarction (MI), history of chronic obstructive pulmonary disease (COPD), and history of thiazide diuretic use.
The Copenhagen City Eye Study 2005 [18]	Denmark	60-80	946 women	14	All-cause:<br>Early vs no AMD: **1.26 (1.05–1.52)**<br>Late vs no AMD:1.28 (0.86–1.90)<br>Any vs no AMD:**1.26 (1.06–1.51)**	Wisconsin Age-Related Maculopathy Grading System	Age, gender, smoking status, hypertension, cardiovascular disease, and diabetes mellitus.
BMES 2007 [17]	Australia	> 49	3654	11	49-74 yrs<br>All-cause:<br>Any AMD vs no AMD:**1.6 (1.0-2.4)**<br>Early vs no AMD:**1.5 (1.0–2.2)**	International Classification System	Age, qualifications, body mass index, smoking status, alcohol consumption, poor self-rated health, walking disability, presence of hypertension and/or diabetes, doctor-diagnosed history of cancer, angina, stroke

© 2018 Zhu Z et al. *JAMA Ophthalmology.*

					Late vs no AMD:**2.5 (1.2–5.0)**		and/or acute myocardial infarction.
					CVD-cause:<br>Any AMD vs no AMD:1.4 (0.6-3.1)<br>Early vs no AMD:1.4 (0.7–2.7)<br>Late vs no AMD:**3.80 (1.4-10.4)**		
BMES 2016 [19]	Australia	> 49	3654	15	All-cause:<br>Any AMD vs no AMD:1.07 (0.87 – 1.32)<br>Early vs no AMD:1.02 (0.80 – 1.31)<br>Late vs no AMD:1.08 (0.79 – 1.49)<br>CVD-cause:<br>Any AMD vs no AMD:0.95 (0.73–1.25)<br>Early vs no AMD:0.82 (0.58 – 1.16)<br>Late vs no AMD:1.20 (0.82 – 1.75)	International Classification System	Age, sex, qualifications, body mass index, smoking status, alcohol consumption, poor self-rated health, walking disability, presence of hypertension and/or<br>diabetes, doctor-diagnosed history of cancer, angina, stroke and/or acute myocardial infarction.
Beaver Dam Eye Study 2012 [16]	USA	43-84	4926	20	All-cause:<br>Late vs no AMD:**1.37 (1.15-1.62)**	Wisconsin Age-Related Maculopathy Grading System	Age and sex.

© 2018 Zhu Z et al. *JAMA Ophthalmology.*

SiMES 2015 [22]	Singapore	40-79	3280	7.24	All-cause:<br>Early vs no AMD:1.08 (0.71-1.55)<br>Late vs no AMD:0.82 (0.34-1.80)	Wisconsin Age-Related Maculopathy Grading System	Age, gender, socio-economic status, hypertension, smoking, BMI and cardiovascular disease.
Beaver Dam Eye Study 2006 [20]	USA	43-84	4926	13.2	All-cause:<br>AMD per 1-step increase in severity: 0.97 (0.87-1.07)	Wisconsin Age-Related Maculopathy Grading System	Age, sex, proteinuria, history of cancer, BMI, BMI2, ratio of total to high-density lipoprotein cholesterol level, smoking, pulse rate, diabetes status, cardiovascular disease history, sedentary lifestyle, education, and systolic blood pressure.
Beijing Eye Study 2008 [24]	China	≥ 40	4439	5	All-cause:<br>Early vs no AMD:1.10 (0.43-2.84)<br>Late vs no AMD:2.81 (0.36-21.89)	International Classification and Grading System	Age and hyperopic refractive error.
VIP 2000 [25]	Australia	≥ 40	3271	5	All-cause:<br>Present vs absent:1.36 (0.96-1.94)	International Classification and Grading System	Age, sex, duration of smoking, duration of high-blood pressure, and arthritis.
APEDS 2013 [40]	Indian	≥ 30	4188	11	All-cause:<br>Any cataract and AMD:1.26 (0.80-1.98)<br>Lens morphology and AMD:1.25 (0.79-1.97)		

© 2018 Zhu Z et al. *JAMA Ophthalmology.*

The Rotterdam Study 2003 [21]	USA	≥ 55	6339	7.0	All-cause:<br>Early vs no AMD:0.96 (0.71-1.29)<br>Late vs no AMD:0.94 (0.52-1.68)	International Classification and Grading System	Age, gender, atherosclerosis, hypertension, diabetes mellitus, smoking, history of cardiovascular disease, body mass index, and cholesterol level.
The United Kingdom 2005 [23]	UK	≥ 75	3201	6.1	All-cause:<br>Late vs no AMD:1.01(0.81-1.25)<br>CVD-cause:<br>Late vs no AMD:1.03 (0.72–1.45)	Glasgow acuity cards, questionnaire and the Ophthalmologist rank	Age and sex.
The Atherosclerosis Risk in Communities Study 2007 [41]	USA	49-73	12536	10	All-cause:<br>Early vs no AMD:0.95 (0.73-1.31)	Wisconsin Age-Related Maculopathy Grading System	Age, sex, race, and field center, education, systolic blood pressure, diastolic blood pressure, diabetes, all-cause plasma cholesterol, triglyceride, glucose, high-density lipoprotein cholesterol, pack-years of cigarette smoking, and current alcohol use.

Abbreviations: AREDS, Age-Related Eye Disease Study; AREDS2, Age-Related Eye Disease Study 2; AGES, The Age, Gene/Environment Susceptibility Reykjavik Study; SOF, Study of Osteoporotic Fractures; BMES, Blue Mountains Eye Study; SiMES, Singapore Malay Eye Study; VIP, Visual Impairment Project; BMI, body mass index; CVD, cardiovascular disease; GA, geographic atrophy.

© 2018 Zhu Z et al. *JAMA Ophthalmology.*
